# Association between risk perception and influenza vaccine hesitancy for children among reproductive women in China during the COVID-19 pandemic: a national online survey

**DOI:** 10.1186/s12889-022-12782-0

**Published:** 2022-02-23

**Authors:** Min Du, Liyuan Tao, Jue Liu

**Affiliations:** 1 Department of Epidemiology and Biostatistics, School of Public Health, Peking University, No.38, Xueyuan Road, Haidian District, Beijing, 100191 China; 2 Research Center of Clinical Epidemiology, Peking University Third Hospital, No.49 Huayuan North Road, Haidian District, Beijing, 100083 China; 3 Institute for Global Health and Development, Peking University, No.5 Yiheyuan Road, Haidian District, Beijing, 100871 China; 4 National Health Commission Key Laboratory of Reproductive Health, Peking University, No.38, Xueyuan Road, Haidian District, Beijing, 100191 China

**Keywords:** Vaccine hesitancy, Influenza, China, Reproductive women, COVID-19

## Abstract

**Background:**

In China, the national prevalence of parental influenza vaccine hesitancy (IVH) during the pandemic of coronavirus disease 2019 (COVID-19), and the association between risk perception and parental IVH are still unclear. We aimed to explore the association between risk perception and IVH for children among reproductive women in China, a poorly studied area.

**Methods:**

From December 14, 2020, to January 31, 2021, we conducted a national anonymous online survey on IVH for children among reproductive women in China. We assessed risk perception including perceived susceptibility, severity, barriers, and benefits using the Health Belief Model and then classified each variable into three groups based on tertiles. Logistic regression models were used to calculate the adjusted odds ratio (aOR) of risk perception related to vaccine hesitancy after controlling for sociodemographic characteristics, health status, and knowledge of influenza, among other factors. Additionally, subgroup analysis was performed.

**Results:**

Among 3,011 reproductive women, 9.13% reported IVH. In multivariable models, vaccine hesitancy was associated with low perceived susceptibility (aOR = 2.55, 95% CI: 1.79–3.65), higher perceived barriers (moderate: aOR = 1.47, 95% CI: 1.04–2.08; high: aOR = 2.20, 95% CI: 1.47–3.30), and low perceived benefit (moderate: aOR = 1.40, 95% CI: 1.03–1.92; low: aOR = 2.10, 95% CI: 1.43–3.07). Subgroup analysis showed that vaccine hesitancy was more likely to occur among women with high perceived barriers aged < 30 years compared with those older than 30 years (P for difference = 0.041) and among women with moderate perceived benefit who had never conceived compared with those had a history of pregnancy (P for difference = 0.048).

**Conclusions:**

Nearly one in 10 reproductive women was hesitant about influenza vaccination for their children during the COVID-19 pandemic. To mitigate vaccine hesitancy, our findings highlight a need for tailored public health measures to increase perceived disease susceptibility and vaccine benefit and decrease perceived barriers. Furthermore, the effect of high perceived barriers and moderate perceived benefit on vaccine hesitancy was higher among younger women and women who had never conceived.

**Supplementary Information:**

The online version contains supplementary material available at 10.1186/s12889-022-12782-0.

## Background

Influenza is an acute respiratory infectious disease that causes three to five million cases of severe illness annually and 290,000–650,000 annual deaths globally [1]. In addition, influenza has high hospitalization rates, as well as substantial morbidity and mortality among children [2]. Influenza vaccination is currently considered the most economical and effective way to prevent this disease [3]. Despite the wide availability and clear vaccination guidelines of influenza vaccines, vaccine uptake remains low among children in most countries, including China [4, 5]. A meta-analysis including 126 studies showed that influenza vaccine coverage is less than 30% among children in mainland China [6]. The estimated five-year average influenza vaccination coverage among children is 5.5% in Shanghai, China [7].

According to the World Health Organization Strategic Advisory Group of Experts, vaccine hesitancy is defined as a delay in acceptance or refusal of vaccination despite the availability of vaccination services [8]. Vaccine hesitancy was declared one of the 10 leading threats to global health in 2019 [9]. As important targets of vaccination, children must rely on parental guidance for decision-making. Thus, decreasing parental influenza vaccine hesitancy (IVH) is critical for improving vaccination coverage among children [10]. In Saudi Arabia in 2019, 23.2% of parents were hesitant regarding vaccinating their children against seasonal influenza [11]. In the United States, the rate of parental IVH was 25.8% in 2018 and 19.5% in 2019 [12]. In China, 24.9% of parents reported hesitancy regarding vaccinating their children with any of the recommended vaccines in 2019 [13]. Wei et al. reported that 43.2% of guardians had IVH in an eastern Chinese province in 2019 [14].

Parental vaccine decision-making is driven by numerous factors, including risk perception (such as perceived risk, utility, and social benefit), lifestyle (such as smoking, drinking, and physical activity), knowledge about vaccines, parental education, household living conditions, and income [15, 16]. Risk perception is a subjective construction process consisting of multiple dimensions, such as judgments regarding the severity and controllability of risks [17]. Flood et al. found that perceptions of susceptibility and the severity of influenza were associated with vaccine hesitancy for children [18]. However, the link with perceived severity of the illness is tenuous and needs more research to be confirmed [19]. Based on cognitive appraisal theory, people may have different risk perceptions regarding potential or actual consequences and the uncontrollability of the COVID-19 pandemic [16, 20]. Additionally, because there are similar initial symptoms between influenza and COVID-19, interest in a safe and effective influenza vaccine may increase [7, 21]. Compared with the pre-pandemic period, willingness to receive influenza vaccines for children increased significantly following the COVID-19 epidemic (78.4% vs. 64.8%) in Shanghai, China [7].

In China, the national prevalence of parental IVH during the COVID-19 pandemic and the association of risk perception on parental IVH are still unclear. Serious challenges in China including a rapidly aging population, as well as the two-child policy and even three-child policy, which have highlighted the importance of education in maternal and child health (MCH) [22]. MCH education on topics such as pre-marital and pre-pregnancy examinations should be disseminated to women of childbearing age, which could help them to possess the knowledge and skills needed to raise future generations [22]. Reproductive-age women are the key target population for MCH education. Therefore, based on a nationally representative sample of reproductive women in China, we aimed to estimate the prevalence of IVH and examine the association between risk perception and vaccine hesitancy for children after controlling for sociodemographic characteristics, health status, and knowledge about influenza.

## Methods

### Study population and design

In this nationwide anonymous cross-sectional survey, we used a stratified random sampling method via an online survey company established in 2006 (Wen Juan Xing; Changsha Ranxing Information Technology Co., Ltd., Hunan, China) from December 14, 2020, to January 31, 2021. As a specialized data science company, Wen Juan Xing [23] has a database comprising factual and well-characterized personal information (e.g., sex, region, and age) for over 2.6 million Chinese respondents. We used this platform to conduct stratified random sampling, recruit target participants, and distribute questionnaire surveys. Based on information recorded in the database, the platform has been used by many researchers in cross-sectional studies to collect data regarding health-related attitudes among the general population [24–26].

We recruited targeted participants in China for this study according to the following inclusion criteria: (1) women aged 18–49 years, and (2) women who agreed to participate in the study. We planned to recruit 3,000 participants. Sampling of participants was divided into three stages via the online survey platform (Wen Juan Xing). First, we divided targeted participants into three regions (eastern, central, and western regions) and randomly selected two provinces from each region. Second, the sample size for each province was allocated in proportion to the population of each province, according to the China Statistical Yearbook 2020 [27]. Third, we used the Wen Juan Xing online platform to randomly select and recruit target participants from the sample database according to the sample size requirements. This study was approved by the Ethical Committee of Peking University Third Hospital (IRB00006761-M2020528) and conducted according to the Declaration of Helsinki. Informed consent was obtained from all participants.

### Assessment of risk perception

We estimated risk perception regarding influenza vaccination with a survey tool commonly used in previous studies for vaccination intention based on the Health Belief Model (HBM), which has good internal consistency and reliability [28, 29]. The HBM is an appropriate theoretical framework for understanding vaccination intent and illustrating the factors influencing people’s decision-making about vaccination [18, 28, 29].

The HBM covers five dimensions and includes nine questions—two on the dimension of “cues to action” and seven on risk perception, including perceived susceptibility, severity, barriers, and benefits. Thus, we used two questions to evaluate perceived susceptibility to infection for the participants themselves and for their children (when applicable), one question to evaluate the perceived severity of infection, three questions to evaluate perceived barriers to vaccination (vaccine safety, effectiveness, and the possibility of infection after vaccination), and one question to evaluate the perceived benefits of vaccination (protective effect). Each question was answered on a three-point Likert scale (“very concerned or agree,” “concerned or not sure,” and “not concerned or disagree,” assigned a score of 3, 2, and 1 points, respectively). The participants were categorized into three groups based on the summed scores for each HBM dimension by tertiles, with the top 33.3% of participants denoted the “high” group, the bottom 33.3% denoted the “low” group, and the middle denoted the “moderate” group. Generally, a Cronbach’s alpha coefficient between 0.6 and 0.8 indicates good internal consistency. In our study, the Cronbach’s alpha index for the different dimensions for influenza vaccination ranged from 0.77 to 0.82, which showed adequate internal consistency and reliability [30].

### Measurement of vaccine hesitancy for children

The primary outcome was parental attitudes toward influenza vaccination for their children. If participants answered “no” to the question “If you have children under 18 years old and a vaccine becomes available, would you be willing to have your children receive the seasonal influenza vaccine?”, they were classified into the hesitancy group.

### Covariates

Except for HBM and attitudes regarding influenza vaccination, the structured self-administered online questionnaire also included items on sociodemographic characteristics, health status, and knowledge about influenza.

Sociodemographic characteristics included age, residential region, education level, occupation, and monthly household income per capita (RMB). Health status covered gravidity, parity, history of chronic disease, and history of influenza vaccination. We evaluated knowledge about influenza in terms of six aspects: source of infection, route of transmission, susceptible populations, common symptoms, high-risk populations for severe illness and death, and individual preventive measures against infection. Each respondent received one point for each correct answer; no points were received for incorrect answers. We divided the total knowledge score into three groups (low, moderate, and high) by tertiles.

### Data analysis

We presented continuous and categorical variables as mean (standard deviation, SD) or percentage (%). The characteristics of participants with vaccine hesitancy were compared using Pearson’s χ2 test. We used univariate and multivariable logistic regression models to estimate the crude odds ratios and adjusted odds ratios (aORs) of vaccine hesitancy in different risk perception groups. A sensitivity analysis was carried out by fitting different models to examine the robustness of the estimation. Model A was a univariate model. In model B, we adjusted sociodemographic characteristics. In model C, we adjusted all covariates, including sociodemographic characteristics, health status, and knowledge of influenza.

Subgroup analyses were performed on each covariate after adjusting the other covariates. The heterogeneity test was used to examine differences between groups, wherein a *P*-value of less than 0.05 indicated a statistically significant difference. All analyses were conducted using IBM SPSS version 25.0 (IBM Corp., Armonk, NY, USA), R version 3.4.0 (The R Project for Statistical Computing, Vienna Austria), and Stata version 16.0 (StataCorp LLC, College Station, TX, USA).

## Results

### Characteristics of participants

Among 3,213 recruited participants, 3,150 completed the questionnaire (the rate of questionnaire completion was 98.04%). We excluded 139 participants because of their extremely short time completing the questionnaire (< 1 min). Ultimately, our study included a total of 3,011 eligible reproductive-age women.

Of the 3,011 included women, 41.35% lived in central China, 23.65% were 21–25 years old, and 54.83% had a bachelor’s degree. The mean scores for perceived susceptibility, severity, barriers, and benefits were 4.06 (SD = 1.23), 2.44 (SD = 0.66), 5.3 (SD = 1.47), and 2.28 (SD = 0.68), respectively. Thus, among 3,011 reproductive women during the COVID-19 epidemic, 43.44%, 53.34%, 49.25%, and 45.73% had moderate perceived susceptibility, high perceived severity, moderate perceived barriers, and moderate perceived benefits, respectively (Table 1).

**Table 1:** Risk perception of 3,011 reproductive women during the COVID-19 epidemic

Risk Perception	N/Mean	%/SD
**Perceived susceptibility**	4.06	1.23
Low	791	26.27%
Moderate	1308	43.44%
High	912	30.29%
**Perceived severity**	2.44	0.66
Low	283	9.40%
Moderate	1122	37.26%
High	1606	53.34%
**Perceived barriers**	5.3	1.47
Low	950	31.55%
Moderate	1483	49.25%
High	578	19.20%
**Perceived benefit**	2.28	0.68
Low	397	13.18%
Moderate	1377	45.73%
High	1237	41.08%

Characteristics of the study population were provided in Table 2. The prevalence of parental IVH among 3,011 reproductive women was 9.13%. According to χ^2^ tests, there were no differences in income for the perceived severity groups. However, vaccine hesitancy was more likely to be observed in women who lived in eastern China, were more than 45 years, had an educational level below high school, had a history of more than two pregnancies, had more than two children, had chronic diseases, were never vaccinated against influenza, and had a low score for knowledge about influenza. Additionally, vaccine hesitancy was more frequent in women with low perceived susceptibility, low perceived benefit, and high perceived barriers (all *P* < 0.05).

**Table 2:** Influenza vaccine hesitancy for children among 3,011 reproductive women in China during the COVID-19 epidemic

Characteristics	N	Vaccine hesitancy for children among women (%)	χ^2^	*P*
**Total**	3,011	275 (9.13)		
**Sociodemographic characteristics**				
**Region**			22.259	< 0.0001
Eastern	920	118 (12.83)		
Central	1,245	98 (7.87)		
Western	846	59 (6.97)		
**Age group (years)**			38.239	< 0.0001
≤ 20	543	35 (6.45)		
21–25	712	44 (6.18)		
26–30	583	53 (9.09)		
31–35	469	48 (10.23)		
36–40	322	32 (9.94)		
41–45	207	33 (15.94)		
> 45	175	30 (17.14)		
**Education**			16.91	< 0.001
Less than high school	321	45 (14.02)		
High school or some college	886	81 (9.14)		
Bachelor’s degree	1,651	128 (7.75)		
Postgraduate degree	153	21 (13.73)		
**Monthly household income per capita (RMB)**			6.215	0.102
≤ 3,000	1562	124 (7.94)		
3,001–5,000	693	68 (9.81)		
5,001–10,000	571	62 (10.86)		
> 10,000	185	21 (11.35)		
**Health status**				
**Gravidity**			11.07	0.004
0	1,607	123 (7.65)		
1	624	60 (9.62)		
≥ 2	780	92 (11.79)		
**Parity**			11.306	0.004
0	1,624	122 (7.51)		
1	825	89 (10.79)		
≥ 2	562	64 (11.39)		
**Chronic disease**			5.01	0.025
Yes	121	18 (14.88)		
No	2,890	257 (8.89)		
**History of influenza vaccination**			33.74	< 0.0001
Yes	833	35 (4.20)		
No	2,178	240 (11.02)		
**Knowledge score**			56.942	< 0.0001
Low	819	123 (15.02)		
Moderate	1,619	131 (8.09)		
High	573	21 (3.66)		
**Risk perception**				
**Perceived susceptibility**			44.518	< 0.0001
Low	791	118 (14.92)		
Moderate	1,308	100 (7.65)		
High	912	57 (6.25)		
**Perceived severity**			4.907	0.086
Low	283	35 (12.37)		
Moderate	1,122	106 (9.45)		
High	1,606	134 (8.34)		
**Perceived barriers**			21,.322	< 0.0001
Low	950	54 (5.68)		
Moderate	1,483	152 (10.25)		
High	578	69 (11.94)		
**Perceived benefit**			29.27	< 0.0001
Low	397	60 (15.11)		
Moderate	1,377	136 (9.88)		
High	1,237	79 (6.39)		

### Association between risk perception and IVH for children

Models A, B, and C were established using logistic regression models, as shown in Table 3. In model A, without controlling for confounding factors, vaccine hesitancy for children was associated with low perceived susceptibility (*P* < 0.0001), low perceived severity (*P* = 0.0298), higher perceived barriers (moderate: *P* < 0.001; high: *P* < 0.0001), and lower perceived benefit (moderate: *P* = 0.0013; low: *P* < 0.0001). After controlling for sociodemographic characteristics in model B, perceived severity was not associated with vaccine hesitancy; the other associations were stable. After controlling all covariates, vaccine hesitancy was associated with low perceived susceptibility (aOR = 2.55, 95% confidence interval [CI]: 1.79–3.65; *P* < 0.0001), higher perceived barriers (moderate: aOR = 1.47, 95% CI: 1.04–2.08, *P* < 0.001; high: aOR = 2.20, 95% CI: 1.47–3.30, *P* < 0.0001), and lower perceived benefit (moderate: aOR = 1.40, 95% CI: 1.03–1.92, *P* = 0.0013; low: aOR = 2.10, 95% CI: 1.43–3.07, *P* < 0.0001).

**Table 3:** Association between risk perception and influenza vaccine hesitancy for children among 3,011 reproductive women in China during the COVID-19 epidemic

Risk perception	Model A	*P*-value	Model B	*P*-value	Model C	*P*-value
	Odds ratio (95% CI)		Adjusted odds ratio (95% CI)		Adjusted odds ratio (95% CI)	
**Perceived susceptibility**						
Low	2.63 (1.89, 3.67)	< 0.0001	2.62 (1.87, 3.68)	< 0.0001	2.55 (1.79, 3.65)	< 0.0001
Moderate	1.24 (0.89, 1.74)	0.2078	1.28 (0.91, 1.80)	0.1548	1.37 (0.96, 1.95)	0.0802
High	Reference		Reference		Reference	
**Perceived severity**						
Low	1.55 (1.04, 2.30)	0.0298	1.45 (0.97, 2.18)	0.0717	1.08 (0.70, 1.67)	0.7306
Moderate	1.15 (0.88, 1.50)	0.3169	1.12 (0.86, 1.47)	0.4013	0.89 (0.67, 1.19)	0.4466
High	Reference		Reference		Reference	
**Perceived barriers**						
Low	Reference		Reference		Reference	
Moderate	1.89 (1.37, 2.61)	0.0001	1.88 (1.36, 2.61)	0.0001	1.47 (1.04, 2.08)	0.0001
High	2.25 (1.55, 3.26)	< 0.0001	2.45 (1.68, 3.59)	< 0.0001	2.20 (1.47, 3.30)	< 0.0001
**Perceived benefit**						
Low	2.61 (1.83, 3.73)	< 0.0001	2.73 (1.89, 3.93)	< 0.0001	2.10 (1.43, 3.07)	0.0002
Moderate	1.61 (1.20, 2.14)	0.0013	1.68 (1.25, 2.25)	0.0005	1.40 (1.03, 1.92)	0.0324
High	Reference		Reference		Reference	

**Fig. 1:**
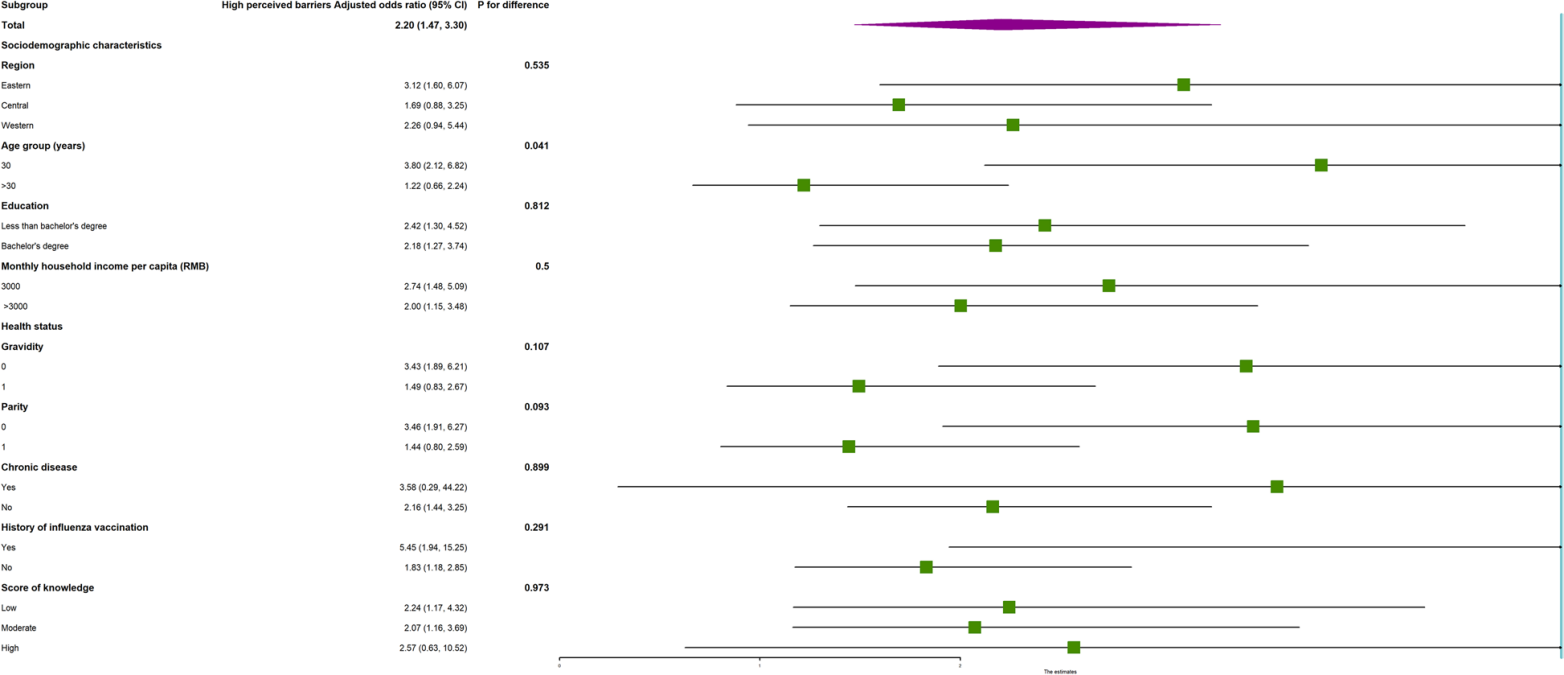
Subgroup analysis of the association between risk perception and vaccine hesitancy for children among reproductive women with high perceived barriers

**Fig. 2:**
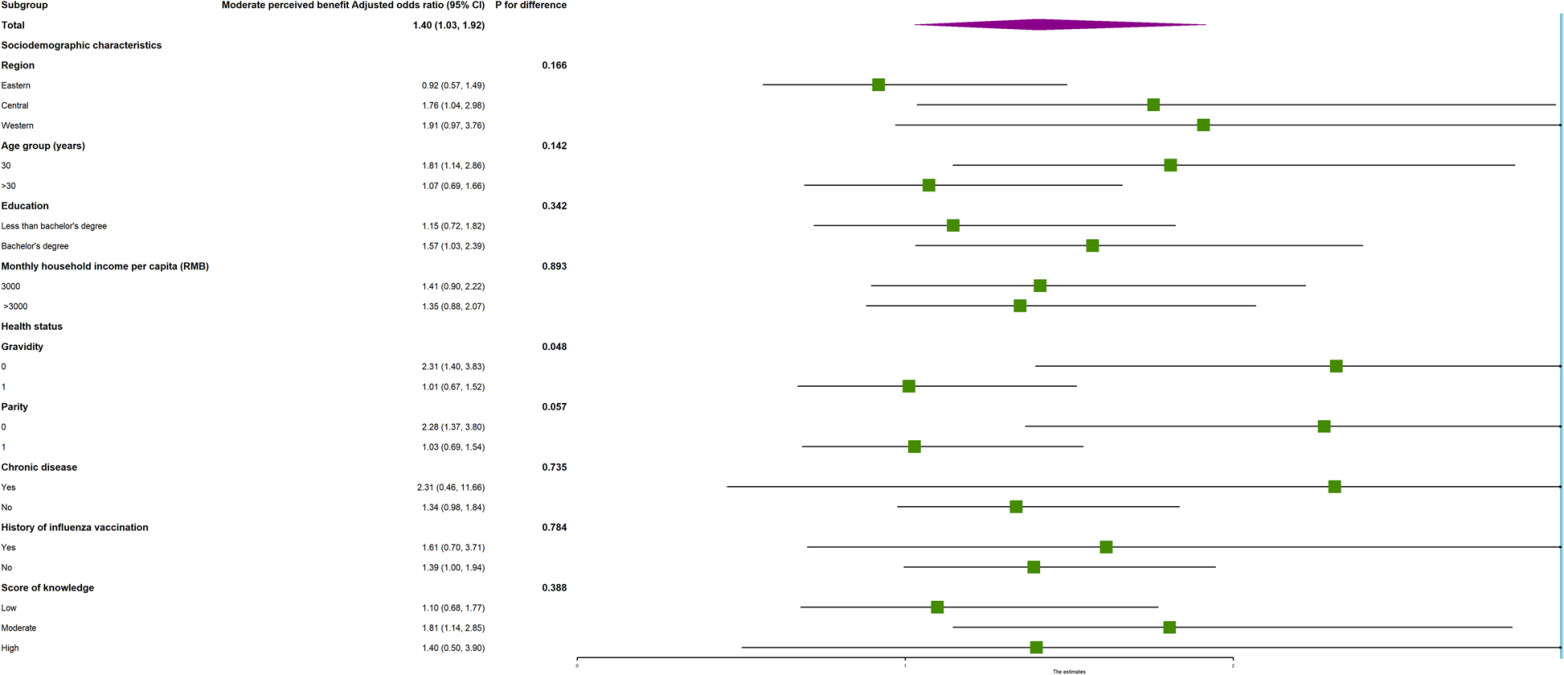
Subgroup analysis of the association between risk perception and vaccine hesitancy for children among reproductive women with moderate perceived benefits

Subgroup analysis showed no interactions in most subgroups (Supplemental Table 1). Nevertheless, for perceived barriers, vaccine hesitancy was more likely to occur among women with high perceived barriers who were 30 years old or less (aOR = 3.80; 95% CI: 2.12–6.82) compared with those older than 30 years (aOR = 1.22; 95% CI: 0.66–2.24) (*P* for difference = 0.041, as shown in Fig. 1). For perceived benefit, vaccine hesitancy was more likely to occur among women with moderate perceived benefit who had never conceived (aOR = 2.31; 95% CI: 1.40–3.83) compared with those who had a history of pregnancy (aOR = 1.01; 95% CI: 0.67–1.52) (*P* for difference = 0.048, as shown in Fig. 2).

## Discussion

To our knowledge, this was the first national online survey to examine the association between risk perception and IVH among reproductive women in China during the COVID-19 pandemic. We found that vaccine hesitancy for children was associated with low perceived susceptibility, higher perceived barriers, and lower perceived benefit, but not with perceived severity.

Although studies on the association between risk perception and vaccine hesitancy for children in China during the COVID-19 pandemic are lacking, our study findings are in line with related studies. Using hypothetical influenza pandemic data, Tan et al. found that among 1,990 respondents, those who exhibited high-risk perception (OR = 1.27, 95% CI = 1.16–1.39) were more likely to have the intention to receive vaccination [31]. Our study showed that vaccine hesitancy for children was associated with low perceived susceptibility, higher perceived barriers, and lower perceived benefit. Flood et al. found that perception of susceptibility to influenza was associated with vaccine hesitancy for children [18]. Madewell et al. found that perceived benefits for mother and infant, and easy accessibility of vaccines were the most cited reasons for influenza vaccination in Honduras [32]. Mills et al. reported that the most common response of parents refusing vaccination was a fear of long-term complications for their children due to vaccines [33]. Our study found that perceived severity was not associated with vaccine hesitancy for children, which was not in accordance with the findings of Flood et al. [18]. However, Smith et al. considered that an association between perceived susceptibility to an illness and child vaccination was strong, but the link with perceived severity of the illness was tenuous [19]. Our results confirmed the opinions of Smith et al. In addition, we found that age and gravidity modified the association of risk perception with vaccine hesitancy for children among women with high perceived barriers and moderate perceived benefit. The effect of high perceived barriers and moderate perceived benefit on vaccine hesitancy was higher among younger women and women who never conceived. Taken together, our findings suggested that to reduce the prevalence of vaccine hesitation for children, national organizations should focus on improving risk perception, including perceived susceptibility, perceived barriers, and perceived benefits, especially for younger women and women who have never conceived.

In our study, only 30.29%, 31.55%, and 41.08% of respondents had high perceived susceptibility, low perceived barriers, and high perceived benefits, respectively. These findings suggest that it is critical to take measures to address IVH such as establishing programs to increase perceived susceptibility and benefit and decrease perceived barriers among parents. Therefore, health care providers should emphasize the benefits of vaccination, vaccine safety and efficacy, and correct misconceptions to address parental concerns regarding the efficacy side effects of vaccination using approaches such as face-to-face education sessions or messages delivered via prerecorded voice message, mail or text [11, 15, 34]. Interventions and training for health care providers are needed to address concerns about the accuracy of professional advice and information sources [35]. Providers, pediatricians, and pediatric specialists should be encouraged to discuss child influenza vaccines with parents at every opportunity [36]. Knowledge about the effects of vaccination in lowering the risk, duration, and severity of influenza as well as the importance of vaccination in preventing influenza transmission among high-risk populations is positively associated with parents’ decision to vaccinate [37]. Additionally, a concerted effort should be made to utilize public health surveillance systems, expand health education in schools, and provide peer comparison as well as online or electronic information [15, 38].

According to our investigation, the prevalence of IVH for children among reproductive women in China was 9.13%. Wang et al. reported that 47.5% of parents were unwilling to permit their children to receive influenza vaccination in Wuxi, eastern China during the COVID-19 epidemic [39]. Zhou et al. reported that the unwillingness to receive influenza vaccination for children following the COVID-19 epidemic was 21.6% in Shanghai, China [7]. According to a Safety-Net Healthcare System report, between August 1, 2019 and February 28, 2020, 13% of parents reported being vaccine-hesitant in the United States [40]. The prevalence of IVH for children in our study was lower than that in the above studies. We speculate that this may be due to the following reasons. (1) During the COVID-19 epidemic, China disseminated extensive publicity regarding influenza vaccination so as to reduce the risk of overlapping co-infection with influenza and COVID-19. National Immunization Program Technical Working Group published the *Technical guidelines for seasonal influenza vaccination in China (2020–2021) &(2021–2022) and* to improve influenza vaccination coverage during the COVID-19 epidemic and called for the public be vaccinated against influenza [41, 42]. (2) In this study, we investigated IVH for children at the national level whereas most previous studies have only conducted investigations at regional or provincial level. There are differences among studies because of their time and scope, which also affected the effect of publicity. In addition, these differences may be related to many factors, including characteristics of the survey populations and survey methods. The results of this study showed that vaccine hesitancy was more likely to occur among women living in eastern China, aged > 45 years, with an education below high school level, a history of more than two pregnancies and more than two children, with a chronic disease, and those who had never been vaccinated against influenza and who had a low score for knowledge about influenza. Studies show that both caregivers’ influenza vaccine history and knowledge about influenza are strong factors in accepting vaccination against influenza for their children [19, 43, 44]. In contrast, the relationship between education and vaccine hesitancy is inconsistent [8, 43, 44].

### Strengths and limitations

A major strength of this national online survey was our estimation of the association between risk perception and IVH among reproductive women in China during the COVID-19 pandemic, the first study of its kind. Our study also had some limitations. First, the population of reproductive women did not completely represent the attitudes of parents throughout China because some participants had no children. Additionally, although participants were recruited using stratified random sampling from in six provinces of China via an online platform, there was a possibility of selection bias and under-coverage of the sample frame because the database of the online platform used an opt-in recruitment strategy. This meant that the results were not nationally representative and may not be generalizable to all women in China. Finally, the effect of tailored public health measures on vaccine hesitancy was not controlled.

## Conclusions

In summary, nearly one in 10 reproductive women was hesitant about influenza vaccination for their children during the COVID-19 pandemic. Vaccine hesitancy was more likely to be observed among women who lived in eastern China, aged > 45 years, who had an education below the high school level, a history of two pregnancies and more than two children, had a chronic disease, had never received influenza vaccination, and had a low score for knowledge about influenza.

Vaccine hesitancy was associated with low perceived susceptibility, higher perceived barriers, and lower perceived benefit. Furthermore, the effect of high perceived barriers and moderate perceived benefit on vaccine hesitancy was higher among younger women and women who had never conceived. Targeted strategies are needed to decrease the prevalence of vaccine hesitancy. National organizations should focus on developing more effective approaches for increasing perceived susceptibility and benefit and decreasing perceived barriers to address IVH during the COVID-19 epidemic.

## Supplementary Information

**Additional file 1.**

## Data Availability

The datasets used and/or analysed during the current study are available from the corresponding author on reasonable request.

## Abbreviations

COVID-19: Coronavirus Disease 2019
HBM: Health Belief Model
IVH: Influenza vaccine hesitancy
MCH: Maternal and child health
OR: Odds ratio
SD: Standard deviation
