# Supplementary material for: Association between risk perception and influenza vaccine hesitancy for children among reproductive women in China during the COVID-19 pandemic: a national online survey
Source: BMC Public Health. 2022 Feb 23;22:385. doi: 10.1186/s12889-022-12782-0 (PMC8865491; doi:10.1186/s12889-022-12782-0)
Supplement: Supplementary file 1 — Additional file 1. [file 12889_2022_12782_MOESM1_ESM.docx]

**Supplemental Table 1. Subgroup analysis of the association between risk perception and vaccine hesitancy for children among reproductive women.**

| **Subgroup** | **Low perceived susceptibility** | **P for difference** | **Moderate perceived susceptibility** | **P for difference** | **Low perceived severity** | **P for difference** | **Moderate perceived severity** | **P for difference** | **Moderate perceived barriers** | **P for difference** | **High perceived barriers** | **P for difference** | **Low perceived benefit** | | | **P for difference** | **Moderate perceived benefit** | | **P for difference** |  |
| --- | --- | --- | --- | --- | --- | --- | --- | --- | --- | --- | --- | --- | --- | --- | --- | --- | --- | --- | --- | --- |
| **Total** | 2.55 (1.79, 3.65) | | 1.37 (0.96, 1.95) | | 1.08 (0.70, 1.67) | | 0.89 (0.67, 1.19) | | 1.47 (1.04, 2.08) | | 2.20 (1.47, 3.30) | | | | 2.10 (1.43, 3.07) | | | 1.40 (1.03, 1.92) | |  |
| **Sociodemographic characteristics** | | | |  |  |  |  |  |  |  |  |  |  | | |  |  | |  |  |
| **Region** |  | 0.166 |  | 0.174 |  | 0.372 |  | 0.118 |  | 0.76 |  | 0.535 |  | | | 0.225 |  | | 0.166 |  |
| Eastern | 4.96 (2.64, 9.29) | | 2.69 (1.43, 5.06) | | 1.25 (0.60, 2.61) | | 1.42 (0.91, 2.21) | | 1.87 (1.08, 3.21) | | 3.12 (1.60, 6.07) | | | | 1.29 (0.68, 2.43) | | | 0.92 (0.57, 1.49) | |  |
| Central | 1.76 (1.01, 3.06) | | 0.95 (0.55, 1.64) | | 0.70 (0.34, 1.46) | | 0.65 (0.40, 1.06) | | 1.37 (0.78, 2.38) | | 1.69 (0.88, 3.25) | | | | 2.97 (1.61, 5.46) | | | 1.76 (1.04, 2.98) | |  |
| Western | 1.55 (0.73, 3.28) | | 0.88 (0.43, 1.79) | | 1.75 (0.76, 4.05) | | 0.74 (0.39, 1.40) | | 1.47 (0.67, 3.25) | | 2.26 (0.94, 5.44) | | | | 2.57 (1.10, 5.99) | | | 1.91 (0.97, 3.76) | |  |
| **Age group (years)** | | 0.586 |  | 0.862 |  | 0.889 |  | 0.403 |  | 0.238 |  | 0.041 |  | | | 0.918 |  | | 0.142 |  |
| <30 | 2.35 (1.43, 3.88) | | 1.37 (0.85, 2.22) | | 1.09 (0.58, 2.03) | | 0.79 (0.52, 1.18) | | 1.98 (1.15, 3.40) | | 3.80 (2.12, 6.82) | | | | 2.14 (1.23, 3.75) | | | 1.81 (1.14, 2.86) | |  |
| >30 | 2.90 (1.74, 4.83) | | 1.47 (0.87, 2.47) | | 1.16 (0.63, 2.15) | | 1.02 (0.67, 1.54) | | 1.22 (0.76, 1.94) | | 1.22 (0.66, 2.24) | | | | 2.05 (1.19, 3.54) | | | 1.07 (0.69, 1.66) | |  |
| **Education** | | 0.099 |  | 0.15 |  | 0.073 |  | 0.452 |  | 0.304 |  | 0.812 |  | | | 0.784 |  | | 0.342 |  |
| Less than bachelor's degree | 1.92 (1.16, 3.18) | | 1.05 (0.64, 1.71) | | 1.69 (0.95, 2.99) | | 1.05 (0.68, 1.63) | | 1.94 (1.12, 3.37) | | 2.42 (1.30, 4.52) | | | | 1.90 (1.09, 3.31) | | | 1.15 (0.72, 1.82) | |  |
| Bachelor's degree | 3.90 (2.32, 6.56) | | 1.91 (1.14, 3.21) | | 0.65 (0.32, 1.33) | | 0.83 (0.56, 1.22) | | 1.28 (0.81, 2.00) | | 2.18 (1.27, 3.74) | | | | 2.13 (1.24, 3.64) | | | 1.57 (1.03, 2.39) | |  |
| **Monthly household income per capita (RMB)** | | 0.23 |  | 0.451 |  | 0.978 |  | 0.146 |  | 0.21 |  | 0.5 |  | | | 0.241 |  | | 0.893 |  |
| ≤3000 | 2.03 (1.22, 3.39) | | 1.19 (0.73, 1.93) | | 1.12 (0.62, 2.04) | | 0.71 (0.46, 1.09) | | 2.09 (1.20, 3.65) | | 2.74 (1.48, 5.09) | | | | 1.60 (0.90, 2.84) | | | 1.41 (0.90, 2.22) | |  |
| >3000 | 3.27 (1.98, 5.39) | | 1.59 (0.95, 2.65) | | 1.11 (0.58, 2.11) | | 1.11 (0.76, 1.64) | | 1.23 (0.78, 1.94) | | 2.00 (1.15, 3.48) | | | | 2.65 (1.57, 4.47) | | | 1.35 (0.88, 2.07) | |  |
| **Health status** | |  |  |  |  |  |  |  |  |  |  |  |  | | |  |  | |  |  |
| **Gravidity** |  | 0.269 |  | 0.839 |  | 0.855 |  | 0.343 |  | 0.871 |  | 0.107 |  | | | 0.225 |  | | 0.048 |  |
| 0 | | 2.03 (1.19, 3.48) | | 1.34 (0.81, 2.24) | | 1.18 (0.62, 2.27) | | 0.75 (0.48, 1.15) | | 1.61 (0.93, 2.77) | | 3.43 (1.89, 6.21) | | | 2.94 (1.63, 5.30) | | | | 2.31 (1.40, 3.83) | |
| ≥1 | | 3.11 (1.94, 5.01) | | 1.45 (0.89, 2.35) | | 1.09 (0.60, 1.95) | | 1.00 (0.68, 1.47) | | 1.51 (0.96, 2.37) | | 1.49 (0.83, 2.67) | | | 1.67 (0.99, 2.82) | | | | 1.01 (0.67, 1.52) | |
| **Parity** |  | 0.52 |  | 0.818 |  | 0.93 |  | 0.665 |  | 0.982 |  | 0.093 |  | | | 0.153 |  | | 0.057 |  |
| 0 | 2.24 (1.30, 3.85) | | 1.31 (0.78, 2.20) | | 1.10 (0.57, 2.14) | | 0.82 (0.54, 1.27) | | 1.54 (0.89, 2.66) | | 3.46 (1.91, 6.27) | | | | 3.16 (1.75, 5.70) | | | 2.28 (1.37, 3.80) | |  |
| ≥1 | 2.86 (1.79, 4.59) | | 1.43 (0.89, 2.31) | | 1.15 (0.65, 2.04) | | 0.94 (0.64, 1.39) | | 1.53 (0.97, 2.39) | | 1.44 (0.80, 2.59) | | | | 1.58 (0.94, 2.68) | | | 1.03 (0.69, 1.54) | |  |
| **Chronic disease** | | 0.96 |  | 0.952 |  | 0.288 |  | 0.926 |  | 0.717 |  | 0.899 |  | | | 0.733 |  | | 0.735 |  |
| Yes | 2.71 (0.56, 13.03) | | 1.51 (0.24, 9.69) | | 0.23 (0.02, 3.38) | | 1.01 (0.27, 3.69) | | 3.33 (0.53, 20.81) | | 3.58 (0.29, 44.22) | | | | 4.44 (0.67, 29.25) | | | 2.31 (0.46, 11.66) | |  |
| No | 2.55 (1.77, 3.67) | | 1.37 (0.96, 1.96) | | 1.19 (0.77, 1.85) | | 0.92 (0.69, 1.24) | | 1.46 (1.03, 2.07) | | 2.16 (1.44, 3.25) | | | | 1.95 (1.31, 2.88) | | | 1.34 (0.98, 1.84) | |  |
| **History of influenza vaccination** | | 0.851 |  | 0.399 |  | 0.666 |  | 0.431 |  | 0.858 |  | 0.291 |  | | | 0.996 |  | | 0.784 |  |
| Yes | 2.33 (0.83, 6.57) | | 2.37 (0.92, 6.12) | | 0.81 (0.22, 2.99) | | 0.68 (0.30, 1.54) | | 1.65 (0.60, 4.55) | | 5.45 (1.94, 15.25) | | | | 2.10 (0.72, 6.07) | | | 1.61 (0.70, 3.71) | |  |
| No | 2.62 (1.80, 3.82) | | 1.24 (0.85, 1.80) | | 1.14 (0.72, 1.82) | | 0.96 (0.70, 1.30) | | 1.47 (1.01, 2.12) | | 1.83 (1.18, 2.85) | | | | 2.10 (1.39, 3.17) | | | 1.39 (1.00, 1.94) | |  |
| **Score of knowledge** | | 0.063 |  | 0.091 |  | 0.933 |  | 0.965 |  | 0.832 |  | 0.973 |  | | | 0.665 |  | | 0.388 |  |
| Low | 1.36 (0.82, 2.26) | | 0.77 (0.47, 1.28) | | 1.07 (0.57, 2.01) | | 0.88 (0.56, 1.37) | | 1.72 (0.95, 3.11) | | 2.24 (1.17, 4.32) | | | | 1.84 (1.04, 3.25) | | | 1.10 (0.68, 1.77) | |  |
| Moderate | 5.17 (2.87, 9.31) | | 2.43 (1.35, 4.36) | | 1.16 (0.61, 2.22) | | 0.91 (0.61, 1.37) | | 1.33 (0.83, 2.12) | | 2.07 (1.16, 3.69) | | | | 2.47 (1.41, 4.33) | | | 1.81 (1.14, 2.85) | |  |
| High | 4.74 (1.09, 20.63) | | 2.82 (0.69, 11.54) | | 1.97 (0.37, 10.36) | | 1.06 (0.38, 2.93) | | 1.47 (0.50, 4.29) | | 2.57 (0.63, 10.52) | | | | 1.15 (0.22, 5.96) | | | 1.40 (0.50, 3.90) | |  |

^1^effect size was aOR and 95%CI
